# Age, period and cohort analysis of patient dental visits in Australia

**DOI:** 10.1186/1472-6963-14-13

**Published:** 2014-01-10

**Authors:** Xiangqun Ju, David S Brennan, A John Spencer

**Affiliations:** 1 Australian Research Centre for Population Oral Health, The University of Adelaide, South Australia 5005, Australia

**Keywords:** Age, Period, Cohort, Patient visits

## Abstract

**Background:**

Understanding dentists’ capacity to supply dental services over time is a key element in the process of planning for the future. The aim was to identify time trends and estimate age, period and cohort effects in patients’ visits supplied per dentist per year.

**Methods:**

Mailed questionnaires were collected from a random sample of Australian private general practice dentists. The response rates were 73%, 75%, 74%, 71%, 76% and 67% in 1983, 1988, 1993, 1998, 2003 and 2009, respectively. The time trends in the mean number of patient visits supplied per dentist per year (PPY) was described by using a standard cohort table and age-period-cohort analyses applying a nested general linear regression models approach.

**Results:**

The mean number of PPY decreased across most age groups of dentists over the time of study. The age-period model showed that younger dentists (20–29 years) and older dentists (65–74 and 80–84 years) had lower PPY than middle-aged dentists, and the age-cohort model showed higher PPY among earlier cohorts, and lower PPY among more recent cohorts.

**Conclusion:**

The study found a period effect of declining PPY over the observation period. More recent cohorts of dentists provide lower numbers of PPY than earlier cohorts at similar ages, but the provision of PPY among these younger cohorts appeared to be stable as they moved into middle age.

## Background

In Australia the majority of practising dentists work in the private sector (83%), with dental services being provided mainly by general dental practitioners (85%) [1]. Dental services are generally provided on a fee-for-service basis, paid either directly by the individual or indirectly through private insurance.

The capacity of practising dentists to supply dental services has not only been linked with population demographics and oral health status, but also associated with labour force structure and service-mix provided. To measure practice activity provided by dentists, the time measure of the hours per dentist per year (HPY) and the patient measure of the number of patient visits per dentist per hour (PPH) were used to produce the measure of patient visits per dentist per year (PPY = HPY × PPH) as in previous research [1]. PPY was adopted as a key marker of dentist’s capacity to provide services and has shown a decreasing trend over time in Australia [2-4].

Age, period and cohort effects are important considerations when explaining trends in patient visits supplied by practising dentists [5]. Age effects are associated with the passage of time, so change in the number of patient visits by practising dentists related to age over time may help to explain the capacity to provide dental service, for instance, if dentists became less productive as they aged. Period effects can affect all ages simultaneously over time. For example, they can mark the occurrence of a particular historical event, such as the availability of modern high-speed electric dental handpieces. Cohort effects involve changes across groups with the same birth year who experience the same event during the same period. There is a linear dependency between age, period and cohort, because age, period and cohort membership is predicted by any two of the three effects. Therefore, it is difficult to estimate the three separate effects. A nested models approach can be used to estimate and assess the fit of different models [6,7], such as age-period and age-cohort models.

Understanding trends over time in the supply of patient visits and the relationship with labour force age structure and possible cohort effects is important in dental labour force planning, informing current policies and projections of future capacity to supply services. The aim of this study was to identify trends in patients visits supplied by practising dentists in Australia, and estimate age, period and cohort effects in PPY over an observation period spanning 1983 to 2010.

## Method

### Data collection

The data were from the Longitudinal Study of Dentists’ Practice Activity, which is designed to provide estimates of dentist practices and service provision of Australian private general practising dentists over time. The study was approved by the ethics committee of the Australian Institute of Health and Welfare (AIHW).

Details of the methods have been previously described [1,8-10]. Briefly, a random sample of 10% of male and 40% of female dentists was selected from the dental registers for each State or Territory in Australia in 1983–84, 1988–89, 1993–94, 1998–99, 2003–04 and 2009–10 as waves of a longitudinal study. All sampled dentists from previous waves of the study were included again at each successive wave. Sample supplementation of newly registered male and female dentists at each successive wave was also used to add to the sample to ensure representative cross-sectional estimates.

These dentists were surveyed by mailed questionnaire. The practising dentists provided estimates of the number of patients treated per day, and the number of hours per day, days per week and weeks per year spent working. From this information, practice activity measures were calculated as follows:

$$\begin{array}{l} \text{Hours per dentist per year}\;\left(\text{HPY}\right)\\ \;=\left(\text{hours per day}\right)\times \left(\text{days per week}\right)\\ \;\times \left(\text{weeks per year}\right)\\ \text{Patient visits per dentist per hour}\;\left(\text{PPH}\right)\\ \;=\left(\text{patients per day}\right)/\left(\text{hours per day}\right) \end{array}$$

$$\begin{array}{l} \text{Patient visits per dentist per year}\;\left(\text{PPY}\right)\\ \;=\text{HPY}\times \text{PPH} \end{array}$$

### Weighting

The data were weighted prior to analysis. The data were weighted using numbers of private general practising dentists at December 1983 and 1988, with age and sex distributions of dentists from the 1981 and 1986 population censuses of Australia, and dental board registration statistics from 1992, 1994, 2000 and 2009 [1,3,8-10]. The weights adjusted the sample to the age-specific population distribution of male and female dentists in the dentist population.

### Analysis

All sampled dentists were included and the analysis treated the sample as a synthetic cohort as this maintained representative cross-sectional estimates at each point in time rather than restricting analyses only to longitudinal cases. A standard cohort table was produced to provide an initial description of the effects of age, period and cohort by creating 5-year age groups and corresponding 5-year birth cohorts. Age group formed the rows of the table and time period formed the columns, which provided synthetic 5-year birth cohorts in each diagonal running downwards from left to right across the table [5].

General linear regression was applied to estimate mean number of PPY by age, period and cohort factors using SAS statistical software (SAS 9.2). A set of nested models were examined for goodness-of-fit, and F-tests were applied to determine which models were preferred [11]. The details are described below.

The age model was used as a starting point in analysis and followed by age-drift, age-period, age-cohort and age-period-cohort models [12]. The age model was made up of 14 age groups coded as indicator (dummy) variables. The age-drift model consisted of the 14 variables for age (coded from 1 to 14), plus the six time periods (coded from 1 to 6) entered as a continuous variable to model regular trends not ascribed to either period or cohort influence. This variation is referred to as "drift" [6,7,12]. The age-period model consisted of the 14 indicator variables for age, with period entered as indicator variables. The age-cohort model consisted of the 14 indicator variables for age, combined with 17 indicator variables for dentist birth cohort. The age-period-cohort model consisted of the indicator variables for age, period and cohort.

Goodness-of-fit tests were applied for each model and F-tests were used to assess the models. The models with a good fit to data were pursued further to provide a parsimonious explanation of the data.

## Results

The response rates were 73%, 75%, 74%, 71%, 76% and 67% in 1883–84, 1988–89, 1993–94, 1998–99, 2003–4 and 2009–10, respectively.

Table 1 is a standard cohort table which presents age distributions of private general practice dentists by time of data collection. Five-year age groups were coded into 14 categories, combined with five-yearly periods (six years interval for 2003 to 2009), which provided synthetic 5-year birth cohorts of practising dentists in each diagonal running downwards from left to right across the table.

**Table 1:** Number of dentists by age group and time of data collection (unweighted data)

**Age group (years)**	**1983**		**1988**		**1993**		**1998**		**2003**		**2009**		**All**	
	**N**	**(%)**	**N**	**(%)**	**N**	**(%)**	**N**	**(%)**	**N**	**(%)**	**N**	**(%)**	**N**	**(%)**
**20-24**	6	1.7	24	5.2	18	4.2	15	3.3	17	3.3	16	2.6	96	3.4
**25-29**	62	17.8	77	16.7	62	14.6	69	15.4	60	11.8	98	15.6	428	15.2
**30-34**	64	18.3	94	20.4	69	16.2	62	13.8	78	15.3	107	17.1	474	16.8
**35-39**	49	14.0	73	15.8	86	20.2	84	18.7	62	12.2	87	13.9	441	15.6
**40-44**	43	12.3	49	10.6	55	12.9	80	17.8	98	19.2	64	10.2	389	13.8
**45-49**	29	8.3	46	10.0	48	11.3	54	12.0	84	16.5	78	12.4	339	12.0
**50-54**	27	7.7	27	5.9	31	7.3	37	8.2	54	10.6	82	13.1	258	9.1
**55-59**	40	11.5	26	5.6	19	4.5	24	5.4	29	5.7	45	7.2	183	6.5
**60-64**	16	4.6	27	5.9	18	4.2	9	2.0	14	2.8	34	5.4	118	4.2
**65-69**	7	2.0	11	2.4	13	3.1	4	0.9	8	1.6	12	1.9	55	1.9
**70-74**	4	1.2	5	1.1	3	0.7	10	2.2	2	0.4	3	0.5	27	1.0
**75-79**	1	0.3	0	0.0	3	0.7	0	0.0	4	0.8	0	0.0	8	0.3
**80-84**	1	0.3	1	0.2	0	0.0	1	0.2	0	0.0	1	0.2	4	0.1
**85-89**	0	0.0	1	0.2	0	0.0	0	0.0	0	0.0	0	0.0	1	0.0
**Total**	349	100.	461	100	425	100	449	100	510	100	627	100.	2821	100

Higher numbers of practicing dentists were observed in the 25–29 to 60–64 year age groups. Cell sizes of less than 10 occurred in the youngest (20–24 years) or 65 years and older groups at some points in time. Dental practice activity for these smaller groups should be interpreted with caution.

**Figure 1:**
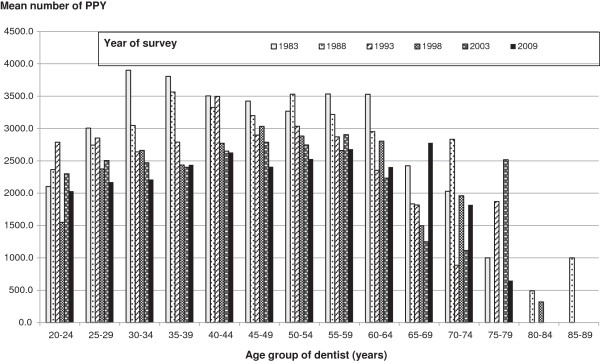
Mean number of PPY by age and year of study.

**Figure 2:**
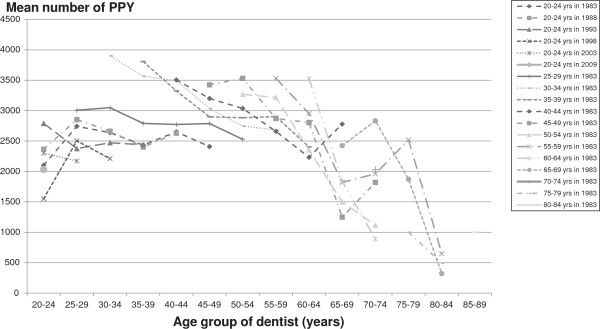
**Mean number of PPY by age and cohort.** Each line represents the mean number of PPY of a dentist birth cohort over time.

Mean number and standard error of PPY by age and year of study are showed in Table 2 and presented graphically in Figures 1 and 2. Figure 1 shows mean number of PPY by age and year of study. In general, mean number of PPY decreased across most age groups over the time of study. PPY was lower in younger age groups (less than 25 years) and older age groups (65 years or older groups), and tended to be higher in middle age groups (30–34 to 60–64 year age groups). Figure 2 shows mean number of PPY by dentist age, year of study and birth cohort. Each line represents a dentist 5-year birth cohort over the times of data collection. Most cohorts are represented by six observation times.

**Table 2:** Mean number and standard error of PPY by age group and time of data collection (weighted)

**Age group (years)**	**1983**		**1988**		**1993**		**1998**		**2003**		**2009**		**All**	
	**Mean**	**SE**	**Mean**	**SE**	**Mean**	**SE**	**Mean**	**SE**	**Mean**	**SE**	**Mean**	**SE**	**Mean**	**SE**
**20-24**	2103	356	2365	163	2789	236	1550	199	2301	262	2033	186	2231	96
**25-29**	3005	151	2743	123	2853	146	2375	111	2505	138	2172	89	2617	53
**30-34**	3902	200	3048	137	2638	155	2662	166	2472	128	2211	113	2772	64
**35-39**	3807	200	3565	177	2790	132	2435	109	2402	152	2440	128	2845	64
**40-44**	3506	238	3326	190	3496	162	2772	144	2652	118	2630	228	2979	73
**45-49**	3425	314	3199	212	2899	173	3033	157	2788	123	2410	128	2859	69
**50-54**	3267	208	3531	332	3036	269	2884	225	2746	154	2529	135	2889	84
**55-59**	3533	303	3218	177	2870	241	2660	305	2904	183	2682	233	3005	106
**60-64**	3529	409	2952	335	2354	171	2805	350	2233	348	2404	156	2603	115
**65-69**	2424	667	1835	257	1818	292	1499	343	1249	273	2779	492	2000	185
**70-74**	2030	466	2833	945	888	168	1961	377	1115	425	1821	1043	1891	256
**75-79**	1000				1871	818			2518	1028	648		2200	602
**80-84**			490				320						476	103
**85-89**			1000										1000	.
**Total**	**3405**	**82**	**3097**	**65**	**2816**	**61**	**2589**	**58**	**2550**	**52**	**2418**	**51**	**2762**	**25**

The fit of a set of age, age-drift, age-period, age-cohort, and age-period-cohort models were assessed for the mean number of PPY. R-squared and P-values from these models are showed in Table 3. A P-value of 0.05 is taken as the significance level, and higher R-squared indicated a better model fit. R-squared increased with more complex models, such as the age drift model’s R-squared was increased over 50% compared to the age model. However, R-squared showed little change from age-drift, age-period or age-cohort models to the age-period-cohort model.

**Table 3:** R-squared and P-values from goodness-of-fit tests from general linear regression analyses

**Models**	**Number**	**Degrees of freedom**	**R squared**	**F value**	**P > F**
**Age**	2776	13	0.039	8.6	<0.0001
**Age-drift**	2776	14	0.099	21.6	<0.0001
**Age-period**	2776	18	0.102	17.4	<0.0001
**Age-cohort**	2776	29	0.111	11.8	<0.0001
**Age-period-cohort**	2776	33	0.115	10.8	<0.0001

F-tests were applied to test age, period and cohort effects in the nested models, which compared the age versus age-drift, then age-drift versus age-period and age-cohort, then age-period and age-cohort versus age-period-cohort models. F and P values from F- tests are presented in Table 4.

**Table 4:** F and P values from F-test and general linear regression analyses

	**Degrees of freedom**			**F value**	**P-value**
**Models**	**k**	**m**	**n-k-m-1**		
**Age & age drift**	13	13	2761	9.66	<0.005
**Age drift & age-period**	14	14	2756	8.88	<0.005
**Age drift & age-cohort**	14	14	2744	13.96	<0.005
**Age-period-Cohort & age-Cohort**	29	29	2741	5.64	<0.005
**Age-period-Cohort & age-Period**	18	18	2740	11.52	<0.005

Notes: **n** was number of observations (see Table 3); **k** was degrees of freedom in the general linear regression model; **m** was additional variables, such as drift, age, period and cohort or their combination.

While the age-period-cohort model provided the best fit to the data, the age, period and cohort effects are not completely independent. The age-period and age-cohort models were therefore examined in order to interpret the effects of age, period and cohort on PPY.

Table 5 presents the parameter estimates and standard error of mean number of PPY from general linear regression for the age-period and age-cohort models. A parameter estimate of 0 indicated which group was the reference group. A parameter estimate less than 0 indicated a lower average number of PPY and greater than 0 indicated higher average number of PPY, compared with the reference group. The reference categories used were the 30–34 year age group, the data collection from 2009–10 and the dentist birth cohort that was aged 25–29 years in 1983.

**Table 5:** Age-period and age-cohort models of PPY

	**Age-period model**			**Age-cohort model**		
	**Parameter estimate**	**SE**	**P**	**Parameter estimate**	**SE**	**P**
**Age group**						
**20-24**	-575.72	167.87	0.0006	-227.75	183.01	0.21
**25-29**	-215.46	91.67	0.0188	21.21	97.33	0.83
**30-34**	0.00			0.00		
**35-39**	81.60	91.10	0.3705	-148.17	94.88	0.12
**40-44**	259.74	92.16	0.0049	-163.53	99.14	0.10
**45-49**	168.54	94.43	0.0744	-447.04	105.24	<.0001
**50-54**	200.62	96.90	0.0385	-623.58	112.21	<.0001
**55-59**	197.47	104.71	0.0594	-811.74	131.32	<.0001
**60-64**	-85.57	114.86	0.4563	-1244.09	144.70	<.0001
**65-69**	-685.31	152.71	<.0001	-2006.21	185.03	<.0001
**70-74**	-805.75	199.77	<.0001	-2291.59	245.17	<.0001
**75-79**	-465.99	341.60	0.1726	-2067.34	392.57	<.0001
**80-84**	-2153.59	546.17	<.0001	-3731.77	620.80	<.0001
**85-89**	-2104.31	1170.04	0.0722	-2054.34	1167.05	0.08
**Period**						
**1983**	993.34	85.61	<.0001			
**1988**	706.21	79.16	<.0001			
**1993**	409.65	80.80	<.0001			
**1998**	188.70	79.83	0.0182			
**2003**	107.99	76.62	0.1588			
**2009**	0.00					
**Cohort**						
**80-84 (a)**	-		-	648.55	958.64	0.50
**75-79 (a)**	-		-	1267.25	686.92	0.07
**70-74 (a)**	-		-	1393.63	356.46	<.0001
**65-69 (a)**	-		-	1223.43	274.77	<.0001
**60-64 (a)**	-		-	1175.36	161.98	<.0001
**50-59 (a)**	-		-	772.51	153.49	<.0001
**45-49 (a)**	-		-	833.53	137.42	<.0001
**40-44 (a)**	-		-	690.22	117.07	<.0001
**35-39 (a)**	-		-	551.33	102.35	<.0001
**30-34 (a)**	-		-	545.69	92.84	<.0001
**25-29 (a)**	-		-	0.00		
**20-24 (a)**	-		-	-330.88	89.43	0.00
**20-24 (b)**	-		-	-352.63	102.26	0.00
**20-24 (c)**	-		-	-539.13	114.43	<.0001
**20-24 (d)**	-		-	-778.79	131.20	<.0001
**20-24 (e)**	-		-	-847.71	162.35	<.0001
**20-24 (f)**				-793.63	455.75	0.08

Note: a = period 1 (1983), b = period 2 (1988), c = period 3 (1993), d = period 4 (1998), e = period 5 (2003), f = period 6 (2009).

In the age-period model, the age effect showed that compared to the reference group of 30–34 years there were negative parameter estimates indicating lower PPY in younger (20–24 and 25–29 years) and older age groups (65–69, 70–74 and 80–84 years). Age groups 40–44 and 50–54 years had positive parameter estimates indicating higher PPY. The period effect showed that compared to the reference group of 2009, the periods 1983 to 1998 had positive parameter estimates indicating higher PPY.

In the age-cohort model, the age effect showed that the age groups 45–49 to 80–84 years all had negative parameter estimates indicating lower PPY than the reference group of 30–34 years. The cohort effect showed that compared to the reference group of 25–29 years in 1983, older cohorts aged 30–34 to 70–74 years in 1983 had positive parameter estimates indicating higher PPY, while younger cohorts aged 20–24 in 1983 to 20–24 years in 2003 had negative parameter estimates indicating lower PPY.

In summary, the cross-sectional age curve from the age-period model shows that the younger age dentists (20–29 years) and older dentists (65–74 and 80–84 years) have lower PPY than middle-aged dentists. The longitudinal age curve from this age-cohort model shows intra-cohort ageing effects of declining PPY over time within cohorts aged 45–84 years. Cohort parameters from the age-cohort model generally show higher PPY among earlier cohorts, and lower PPY among more recent cohorts.

## Discussion

The present study investigated time trends and estimated age, period and cohort effects in patient visits supplied per dentist per year. The findings of the study have shown that the mean number of PPY decreased across most age groups of dentists over time, and the younger age dentists (20–29 years) and older dentists (65–74 and 80–84 years) have lower PPY than middle-aged dentists. There were intra-cohort ageing effects of declining PPY over time within cohorts aged 45–84 years, and higher PPY among earlier cohorts, and PPY was lower among more recent cohorts.

This longitudinal study was from a national survey and samples were selected randomly from a comprehensive sampling frame and achieved about 70 per cent response rates at each wave. Because the majority of dentists in Australia were from general practice and were from the private sector [3,6,13], the data was weighted to reflect the age and sex distribution of private general practitioners in Australia. Therefore, the results can be generalized to represent the main Australian dentist context of private general practice.

These findings point to a fundamentally different pattern of work for younger cohorts of dentists than older dentist cohorts. The findings show that younger cohorts are providing fewer patients visits each working year, and this work pattern appears to be relatively stable over time as they move into middle age. Previous reports have shown that the trend towards fewer patient visits was related to increased provision of services per visit and a shift in the types of services provided [1].

There are many factors that may influence and impact on PPY. The increasing proportion of female dentists (from 10% in 1980 to 33% in 2009) can have a substantial influence on total aggregate capacity to provide dental services. Similarly, the proportion of female dentists increased in some other western developed countries, such as in Canada (from 17% in 1991 to 37% in 2008) [14] and America (from 3% in 1980 to 19% in 2000) [15]. This is because female dentists were undertaking more part time work [3], and taking more career breaks than male dentists [16-18]. The average worked hours per week decreased (from 39 hours in 2000 to 37 hours in 2009). The percentage working in solo private practice decreased (from 44% in 2000 to 29% in 2009) to reflect a more flexible working pattern, such as solo with assistant, partnership and associateship arrangements [3].

Retaining more natural teeth in middle and older aged adults may consequently result in an increased burden of dental disease in older mouths [19] that may lead to demand for dental services. More complex dental treatment needs may lead to increased length of dental appointments, resulting in lower PPY. For instance, more endodontic and crown and bridge services have been associated with trends towards greater retention of teeth among adults [1,19], as well as age-related oral diagnoses and insurance status [20,21].

Age-period-cohort models provide a formal framework to guide the analysis through an explicit consideration of all effects with assessing goodness-of-fit of models. Using the modelling approach to analyse age, period and cohort effects provides information to understand the time trends and inter-related time-dependent variables of age, period and cohort effects.

These findings are important to labour force planning in relation to the capacity to supply dental services. Australia’s National Oral Health Plan included consideration of a sufficient, sustainable and appropriately skilled labour force to meet identified oral health needs across the Australian population [22] while the National Advisory Council on Dental Health conclude that advancement of foundational activities (such as those relating to the dental labour force) was integral to dental services delivery [23]. A review of Australian government health labour force programs noted the importance of data to inform dental policy debate [24]. Previous projections of the dental labour force in Australia have noted the importance of supply of dental visits to capacity to supply services [25]. Health Workforce Australia is investigating the number and mix in the oral health workforce to meet the changing demographics and policy requirements to 2025 [26].

The synthetic cohort approach used in this study was representative in terms of cross-sectional estimates, rather than being based on longitudinal changes. PPY was a key marker of practice activity. However, the component variables of HPY and PPH were not explicitly modelled. For instance, increased numbers of services per visit over time could decrease PPH [1], and an increase in the number of dentists per practice, dental assistants per practice, or the size of private practice (single handed or group) could reduce HPY. The age-period-cohort approach suffers from a confounding of age, period and cohort effects. This confounding makes the separation of age, period and cohort effects difficult unless all comparisons are pronounced and consistent [5]. Despite these limitations, this study of age, period and cohort effects in relation to patient dental visits in Australia is significant to future planning of the dental labour force in Australia.

## Conclusion

The capacity of dentists to supply services might be influenced by age, period and cohort effects. Understanding dentists’ capacity to supply dental services over time is a key element in the process of planning for the future. The study found a period effect of declining PPY over the observation period. More recent cohorts of dentists provided lower numbers of PPY than earlier cohorts at similar ages, but the provision of PPY among these younger cohorts appeared to be stable as they moved into middle age.

## Abbreviations

HPY: Hours per dentist per year; PPH: Patient visits per dentist per hour; PPY: Patient visits per dentist per year.

## Competing interests

The authors have no competing interests to declare.

## Authors’ contributions

XJ performed analyses and drafted the manuscript. DSB was involved in interpretation of data and contributing of drafting of the manuscript. AJS was involved in developing the project and revising the manuscript. The authors have read and approved the manuscript.

## Pre-publication history

The pre-publication history for this paper can be accessed here:

http://www.biomedcentral.com/1472-6963/14/13/prepub
